# Trastuzumab deruxtecan versus trastuzumab emtansine in HER2-positive metastatic breast cancer: long-term survival analysis of the DESTINY-Breast03 trial

**DOI:** 10.1038/s41591-024-03021-7

**Published:** 2024-06-02

**Authors:** Javier Cortés, Sara A. Hurvitz, Seock-Ah Im, Hiroji Iwata, Giuseppe Curigliano, Sung-Bae Kim, Joanne W. Y. Chiu, Jose L. Pedrini, Wei Li, Kan Yonemori, Giampaolo Bianchini, Sherene Loi, Giuliano S. Borges, Xian Wang, Thomas Bachelot, Shunsuke Nakatani, Shahid Ashfaque, Zhengkang Liang, Anton Egorov, Erika Hamilton

**Affiliations:** 1 Quironsalud Group, Pangaea Oncology, International Breast Cancer Center, Madrid, Spain; 2 IOB Madrid, Hospital Beata Maria Ana, Madrid, Spain; 3 Department of Medicine, Faculty of Biomedical and Health Sciences, Universidad Europea de Madrid, Madrid, Spain; 4 Fred Hutchinson Cancer Center, University of Washington School of Medicine, Seattle, WA USA; 5 Seoul National University Hospital, Cancer Research Institute, Seoul National University College of Medicine, Seoul, Republic of Korea; 6 Aichi Cancer Center Hospital, Nagoya, Japan; 7 European Institute of Oncology, IRCCS, Milan, Italy; 8 Department of Oncology and Hemato-Oncology, University of Milano, Milan, Italy; 9 Asan Medical Center, University of Ulsan College of Medicine, Seoul, Republic of Korea; 10 University of Hong Kong, Hong Kong, China; 11 Hospital Nossa Senhora da Conceição, Porto Alegre, Brazil; 12 The First Hospital of Jilin University, Changchun, China; 13 National Cancer Center Hospital, Tokyo, Japan; 14 IRCCS San Raffaele Hospital and Università Vita-Salute San Raffaele, Milan, Italy; 15 Peter MacCallum Cancer Centre, the University of Melbourne, Melbourne, Victoria Australia; 16 Clínica de Neoplasias Litoral, Catarina Pesquisa Clínica, Itajaí, Brazil; 17 Zhejiang University School of Medicine, Sir Run Run Shaw Hospital, Hangzhou, China; 18 Centre Léon Bérard, Lyon, France; 19 Daiichi Sankyo Co, Ltd., Tokyo, Japan; 20 Daiichi Sankyo, Inc., Basking Ridge, NJ USA; 21 Daiichi Sankyo, Inc., Rueil-Malmaison, France; 22 Sarah Cannon Research Institute, Nashville, TN USA

**Keywords:** Targeted therapies, Breast cancer

## Abstract

Trastuzumab deruxtecan (T-DXd) demonstrated significantly improved efficacy over trastuzumab emtansine (T-DM1) in DESTINY-Breast03 (median follow-up, 28 months). We report updated efficacy and safety analyses, including secondary and exploratory efficacy endpoints (median follow-up, 41 months) of DESTINY-Breast03. Patients with advanced HER2-positive metastatic breast cancer previously treated with taxane and trastuzumab were randomized to T-DXd (5.4 mg per kg (261 patients)) or T-DM1 (3.6 mg per kg (263 patients)). The primary endpoint was progression-free survival (PFS) by blinded independent central review and was previously reported. The key secondary endpoint was overall survival (OS). Other secondary endpoints included objective response rate, duration of response and PFS (all by investigator assessment) and safety. At data cutoff, 20 November 2023, median PFS by investigator assessment was 29.0 versus 7.2 months (hazard ratio (HR), 0.30; 95% confidence interval (CI), 0.24–0.38), the 36-month PFS rate was 45.7% versus 12.4% and median OS was 52.6 versus 42.7 months (HR, 0.73; 95% CI, 0.56–0.94) with T-DXd versus T-DM1, respectively. Treatment-emergent adverse events were consistent with the previous analyses. No new instances of grade ≥3 interstitial lung disease or pneumonitis occurred (all grade rate, 16.7% (T-DXd) versus 3.4% (T-DM1)). With longer follow-up, T-DXd continued to demonstrate superior efficacy over T-DM1 with a manageable safety profile. ClinicalTrials.gov registration: NCT03529110.

## Abstract (web summary)

Secondary and selected exploratory endpoints of the DESTINY-Breast03 trial of trastuzumab deruxtecan or trastuzumab emtansine in patients with advanced HER2-positive breast cancer are reported, including updated efficacy and safety data after a median follow-up of 41 months.

## Main

Human epidermal growth factor receptor 2 (HER2)-positive breast cancer is characterized by amplification of the *HER2* (*ERBB2*) gene and/or overexpression of the HER2 protein, which stimulates cell proliferation, survival, differentiation, angiogenesis and invasion^1–5^. High levels of HER2 expression have been reported in approximately 20% of all breast cancer tumors, resulting in a more aggressive subtype that metastasizes at a faster rate than breast tumors that do not overexpress HER2 (refs. ^1,2,5–8^). The discovery of HER2 alterations led to the development of treatments that specifically target HER2, resulting in improved prognosis for patients with this subtype of breast cancer^1,2,7,9–12^.

T-DXd is approved in several regions across the globe, including the United States, the European Union and Japan, for patients with HER2-positive metastatic breast cancer after disease progression on taxane and trastuzumab or in patients who have developed disease recurrence during or within 6 months of completing neoadjuvant and/or adjuvant therapy; T-DXd is now a guideline-recommended treatment^11,13–16^. The approval of T-DXd in this setting was based on the results of DESTINY-Breast03 (NCT03529110), a multicenter phase 3 trial conducted to investigate the efficacy and safety of T-DXd versus T-DM1 (ref. ^13^). Before approval of T-DXd, T-DM1 was primarily used in this setting.

T-DXd and T-DM1 are both antibody–drug conjugates composed of humanized monoclonal antibodies targeting HER2, linked to a cytotoxic payload^17–19^. T-DM1 incorporates a microtubule-disrupting agent, which is tethered by a durable thioether bond, whereas T-DXd employs a topoisomerase I inhibitor connected through a tetrapeptide-based cleavable linker, which enables greater specificity in targeting cancer cells, thereby diminishing unintended toxicity^17,19,20^. T-DXd has a high, homogeneous drug-to-antibody ratio of approximately 8, while T-DM1 has a drug-to-antibody ratio of approximately 3.5 (refs. ^17,19,21^).

The primary endpoint of DESTINY-Breast03 was PFS, as determined by blinded independent central review (BICR), and the key secondary endpoint was OS. In the primary (first interim) analysis (data cutoff, 21 May 2021) of DESTINY-Breast03, the primary endpoint was met, with median PFS not reached for T-DXd compared with 6.8 months for T-DM1 (HR, 0.28; 95% CI, 0.22–0.37; *P* < 0.001)^22^. In the second OS interim analysis (data cutoff, 25 July 2022), T-DXd demonstrated a statistically significant and clinically meaningful OS improvement versus T-DM1, with a reduction in the risk for death of approximately 36% (HR, 0.64; 95% CI, 0.47–0.87; *P* = 0.0037)^23^. However, median OS was not reached in either treatment group at the primary analysis or the second OS interim analysis^22,23^.

After the demonstrated statistically significant improvement of PFS with T-DXd versus T-DM1 in the first interim analysis^22^ and updated analysis of PFS at the time of the second OS interim analysis^23^, further assessment of tumor response by BICR was discontinued. We report on an exploratory analysis of DESTINY-Breast03 (data cutoff, 20 November 2023), with updated efficacy, including median OS, and safety data with longer follow-up.

## Results

### Patients

**Fig. 1:**
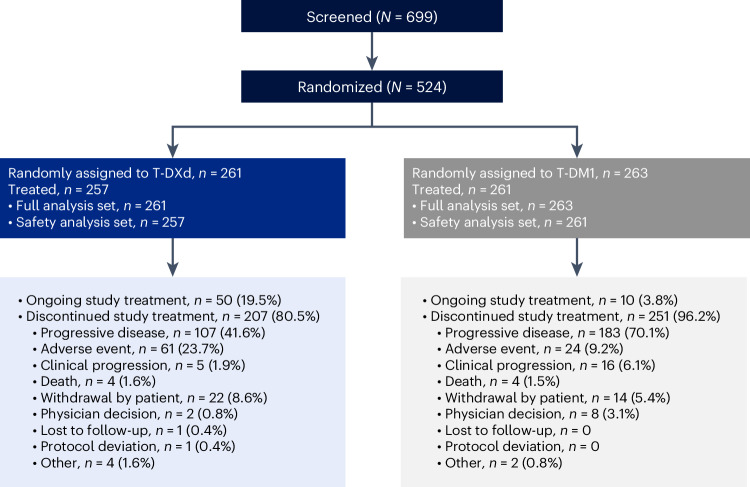
Patient disposition. Efficacy analysis was conducted in the full analysis set (all patients who were randomly assigned to a treatment group), and safety analysis was conducted in the safety analysis set (all patients who were randomly assigned and received at least one dose of T-DXd or T-DM1).

From 20 July 2018 to 23 June 2020, 699 patients were screened for eligibility to enroll in the trial. Five hundred twenty-four patients with HER2-positive, unresectable or metastatic breast cancer were enrolled and randomly assigned 1:1 to receive either T-DXd at 5.4 mg per kg (*n* = 261) or T-DM1 at 3.6 mg per kg (*n* = 263) intravenously once every 3 weeks (Fig. 1). Demographic and baseline characteristics were similar between the two treatment groups (Table 1). The median age was 54.3 years (range, 27.9–83.1 years) in the T-DXd group and 54.2 years (range, 20.2–83.0 years) in the T-DM1 group. An Eastern Cooperative Oncology Group performance score (ECOG PS) of 0 at baseline was reported for 154 patients (59.0%) in the T-DXd group and for 175 patients (66.5%) in the T-DM1 group, whereas 106 patients (40.6%) and 87 patients (33.1%), respectively, had an ECOG PS of 1. In both groups, the majority of patients had a HER2 immunohistochemistry (IHC) score of 3+ (T-DXd, 234 patients (89.7%); T-DM1, 232 patients (88.2%)). Baseline central nervous system (CNS) metastases were reported in 43 patients (16.5%) in the T-DXd group and in 39 patients (14.8%) in the T-DM1 group.

**Table 1:** Patient characteristics, demographics and previous therapies at baseline

Baseline characteristic	T-DXd, 5.4 mg/kg Q3W, *n* = 261	T-DM1, 3.6 mg/kg Q3W, *n* = 263
**Age, median (range), years**	54.3 (27.9–83.1)	54.2 (20.2–83.0)
**Sex,** ***n*** **(%)**		
Female	260 (99.6)	262 (99.6)
Male	1 (0.4)	1 (0.4)
**Region,** ***n*** **(%)**		
Asia	149 (57.1)	160 (60.8)
Europe	54 (20.7)	50 (19.0)
North America	17 (6.5)	17 (6.5)
Australia and South America	41 (15.7)	36 (13.7)
**Race,** ***n*** **(%)**		
White	71 (27.2)	72 (27.4)
Black or African American	10 (3.8)	9 (3.4)
Asian	152 (58.2)	162 (61.6)
Other^a^	28 (10.7)	20 (7.6)
**Ethnicity,** ***n*** **(%)**		
Hispanic/Latino	29 (11.1)	29 (11.0)
Non-Hispanic/non-Latino	203 (77.8)	209 (79.5)
Unknown	5 (1.9)	6 (2.3)
Data not collected	24 (9.2)	19 (7.2)
**HER2 IHC status,** ***n*** **(%)**		
IHC 3+	234 (89.7)	232 (88.2)
IHC 2+	25 (9.6)	30 (11.4)
IHC 1+	1 (0.4)	0
Not evaluable	1 (0.4)	1 (0.4)
***HER2*** **amplification status,** ***n*** **(%)**		
ISH+	24 (9.2)	29 (11.0)
ISH−	2 (0.8)	2 (0.8)
Missing	235 (90.0)	232 (88.2)
**ECOG PS,** ***n*** **(%)**		
0	154 (59.0)	175 (66.5)
1	106 (40.6)	87 (33.1)
Missing	1 (0.4)	1 (0.4)
**Positive hormone receptor status**^**b**^, ***n*** **(%)**	131 (50.2)	134 (51.0)
**History of CNS metastases,** ***n*** **(%)**	62 (23.8)	52 (19.8)
**CNS metastases at baseline,** ***n*** **(%)**	43 (16.5)	39 (14.8)
**Liver metastases at baseline,** ***n*** **(%)**	91 (34.9)	76 (28.9)
**Lung metastases at baseline,** ***n*** **(%)**	113 (43.3)	130 (49.4)
**History of visceral disease,** ***n*** **(%)**	184 (70.5)	185 (70.3)
**Renal impairment at baseline**^**c**^, ***n*** **(%)**		
Within normal range	135 (51.7)	132 (50.2)
Mild impairment	97 (37.2)	105 (39.9)
Moderate impairment	28 (10.7)	25 (9.5)
Missing	1 (0.4)	1 (0.4)
**Any previous systemic cancer therapy**^**d**^, ***n*** **(%)**	260 (99.6)	262 (99.6)
Trastuzumab	260 (99.6)	262 (99.6)
T-DM1	1 (0.4)	0
Pertuzumab	162 (62.1)	158 (60.1)
Taxane and trastuzumab	260 (99.6)	262 (99.6)
Other anti-HER2 therapy	42 (16.1)	38 (14.4)
HER2 TKI	42 (16.1)	36 (13.7)
Other anti-HER2 antibody or antibody–drug conjugate	2 (0.8)	3 (1.1)
Hormone therapy	109 (41.8)	112 (42.6)
Other systemic therapy, not hormone or HER2-directed	183 (70.1)	177 (67.3)
**Number of previous lines of therapy in the metastatic setting, median (range)**	2 (0–16)	2 (0–15)
**Previous treatment for metastatic breast cancer,** ***n*** **(%)**	240 (92.0)	234 (89.0)
**Previous lines of therapy in the metastatic setting**^**e**^, ***n*** **(%)**		
0	1 (0.4)	1 (0.4)
1	108 (41.4)	102 (38.8)
2	60 (23.0)	64 (24.3)
3	44 (16.9)	45 (17.1)
4	15 (5.7)	23 (8.7)
≥5	33 (12.6)	28 (10.6)

CNS, central nervous system; ISH, in situ hybridization; Q3W, every 3 weeks; TKI, tyrosine kinase inhibitor.

^a^Includes patients who reported multiple races.

^b^Patients’ tumors were considered hormone receptor positive if they were estrogen receptor positive and/or progesterone receptor positive.

^c^Within normal range (creatinine clearance ≥90 ml min^−1^), mild impairment (creatinine clearance ≥60 and <90 ml min^−1^) and moderate impairment (creatinine clearance ≥30 and <60 ml min^−1^).

^d^Two patients (one in each treatment group) were randomized in error, and the previous cancer systemic therapy case report form was not completed.

^e^Includes regimens indicated for advanced and/or metastatic disease or early progression within 6 months of regimen for (neo)adjuvant (12 months for pertuzumab).

In both the T-DXd and T-DM1 groups, patients had received a median of two prior lines of therapy in the metastatic setting. As of 20 November 2023, 50 patients (19.5%) in the T-DXd group and ten patients (3.8%) in the T-DM1 group remained on treatment (Fig. 1). The most common reasons patients discontinued study treatment were progressive disease or clinical progression (T-DXd, 107 patients (41.6%) and five patients (1.9%); T-DM1, 183 patients (70.1%) and 16 patients (6.1%)), adverse events (T-DXd, 61 patients (23.7%); T-DM1, 24 patients (9.2%)) and withdrawal by patient (T-DXd, 22 patients (8.6%); T-DM1, 14 patients (5.4%)). Median duration of follow-up was 43.0 months (range, 0.0–62.9 months) for T-DXd and 35.4 months (range, 0.0–60.9 months) for T-DM1.

### Efficacy

**Extended Data Fig. 1:**
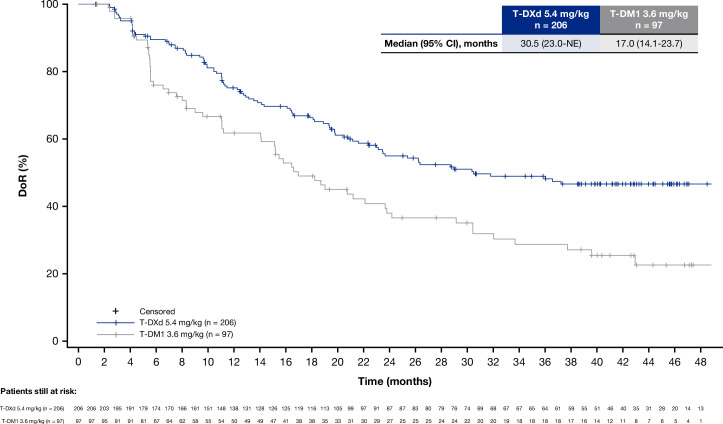
Kaplan-Meier estimates of DoR^a^. DoR, duration of response; NE, not estimable; T-DM1, trastuzumab emtansine; T-DXd, trastuzumab deruxtecan. ^a^The DoR rate at 36 months was 48.9% (95% CI, 41.3-56.1%) with T-DXd and 28.7% (95% CI, 18.9-39.2%) with T-DM1.

The confirmed objective response rate (ORR) by investigator assessment was 78.9% (206 patients; 95% CI, 73.5–83.7%) with T-DXd and 36.9% (97 patients; 95% CI, 31.0–43.0%) with T-DM1 (Table 2). In the T-DXd and T-DM1 groups, respectively, 33 patients (12.6%) and 11 patients (4.2%) experienced a complete response and 173 patients (66.3%) and 86 patients (32.7%) experienced a partial response. The median duration of response (DoR) by investigator assessment was 30.5 months (95% CI, 23.0 months to not estimable (NE)) with T-DXd and 17.0 months (95% CI, 14.1–23.7 months) with T-DM1 (Extended Data Fig. 1).

**Table 2:** Efficacy summary

	T-DXd, 5.4 mg/kg Q3W, *n* = 261	T-DM1, 3.6 mg/kg Q3W, *n* = 263
**OS**^**a**^, **median (95% CI), months**	52.6 (48.7–NE)	42.7 (35.4–NE)
HR (95% CI)	0.73 (0.56–0.94)	
Patients with events (deaths), *n* (%)	110 (42.1)	126 (47.9)
Patients without events (censored), *n* (%)	151 (57.9)	137 (52.1)
Alive	130 (49.8)	112 (42.6)
Lost to follow-up	21 (8.0)	25 (9.5)
OS rate^b^ (95% CI), %		
24 months	77.5 (71.8–82.2)	70.1 (64.0–75.4)
36 months	67.6 (61.3–73.0)	55.7 (49.2–61.7)
48 months	56.9 (50.2–63.1)	48.3 (41.7–54.5)
**PFS**^**a,c**^, **median (95% CI), months**	29.0 (23.7–40.0)	7.2 (6.8–8.3)
HR (95% CI)	0.30 (0.24–0.38)	
Patients with events, *n* (%)	129 (49.4)	197 (74.9)
Progressive disease	120 (46.0)	189 (71.9)
Death	9 (3.4)	8 (3.0)
Patients without events (censored), *n* (%)	132 (50.6)	66 (25.1)
PFS rate^b^ (95% CI), %		
24 months	55.8 (49.1–62.0)	20.6 (15.4–26.4)
36 months	45.7 (38.9–52.2)	12.4 (8.1–17.7)
48 months	41.5 (34.6–48.3)	9.9 (5.9–15.1)
**Confirmed ORR**^**c**^, ***n*** **(%)** **(95% CI)**^**d**^	206 (78.9) (73.5–83.7)	97 (36.9) (31.0–43.0)
Complete response, *n* (%)	33 (12.6)	11 (4.2)
Partial response, *n* (%)	173 (66.3)	86 (32.7)
Stable disease, *n* (%)	48 (18.4)	119 (45.2)
Progressive disease, *n* (%)	2 (0.8)	34 (12.9)
Not evaluable, *n* (%)	5 (1.9)	13 (4.9)
**DoR**^**a,c**^, **median (95% CI), months**	30.5 (23.0–NE)	17.0 (14.1–23.7)
**PFS2**^**a,c**^, **median (95% CI), months**	45.2 (39.3–NE)	23.1 (17.8–29.7)
HR (95% CI)	0.53 (0.41–0.68)	

^a^The median is from Kaplan–Meier analysis. The CI for the median was computed using the Brookmeyer–Crowley method.

^b^Estimate and CI for OS and PFS rates at the specified time points were from Kaplan–Meier analysis.

^c^By investigator assessment.

^d^Based on the Clopper–Pearson method for single proportion and for the difference of two proportions with continuity correction.

**Fig. 2:**
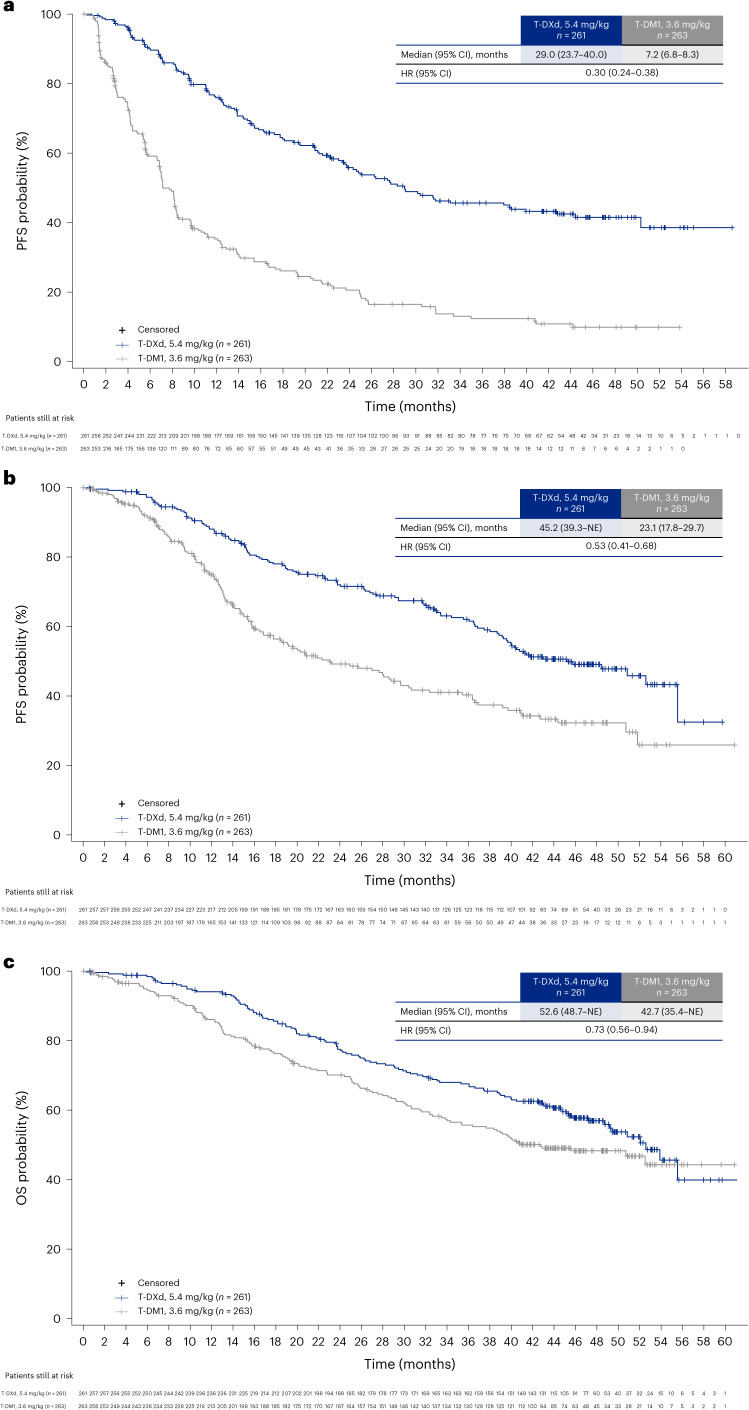
Kaplan–Meier estimates. **a**, PFS. **b**, PFS2. **c**, OS. Crosses indicate where data were censored; numbers of patients censored are not stated.

Median PFS by investigator assessment was 29.0 months (95% CI, 23.7–40.0 months) with T-DXd and 7.2 months (95% CI, 6.8–8.3 months) with T-DM1 (HR, 0.30; 95% CI, 0.24–0.38) (Fig. 2a). The PFS rate at 36 months was 45.7% (95% CI, 38.9–52.2%) with T-DXd and 12.4% (95% CI, 8.1–17.7%) with T-DM1.

In the T-DXd and T-DM1 groups, of the patients who discontinued treatment, 144 patients (69.6%) and 198 patients (78.9%), respectively, received anticancer systemic therapy after the trial (Extended Data Table 1). In the T-DXd group, 75 patients (52.1%) received T-DM1 and 12 patients (8.3%) received T-DXd; in the T-DM1 group, 64 patients (32.3%) received T-DXd and 26 patients (13.1%) received T-DM1 after the trial. Median PFS2 (PFS from the time of randomization to progression on the next line of therapy or death) by investigator assessment was 45.2 months (95% CI, 39.3 months to NE) with T-DXd and 23.1 months (95% CI, 17.8–29.7 months) with T-DM1 (HR, 0.53; 95% CI, 0.41–0.68) (Fig. 2b). The PFS2 rate at 36 months was 62.1% (95% CI, 55.5–68.0%) with T-DXd and 40.3% (95% CI, 33.3–47.2%) with T-DM1.

Two hundred thirty-six OS events were observed up to the data cutoff of 20 November 2023: 110 (42.1%) in the T-DXd group and 126 (47.9%) in the T-DM1 group. Median OS was 52.6 months (95% CI, 48.7 months to NE) with T-DXd and 42.7 months (95% CI, 35.4 months to NE) with T-DM1; the risk of death was reduced by 27% (HR, 0.73; 95% CI, 0.56–0.94) (Fig. 2c and Table 2). The OS rate at 24 months was 77.5% (95% CI, 71.8–82.2%) with T-DXd versus 70.1% (95% CI, 64.0–75.4%) with T-DM1, and the OS rate at 36 months was 67.6% (95% CI, 61.3–73.0%) versus 55.7% (95% CI, 49.2–61.7%), respectively.

**Extended Data Fig. 2:**
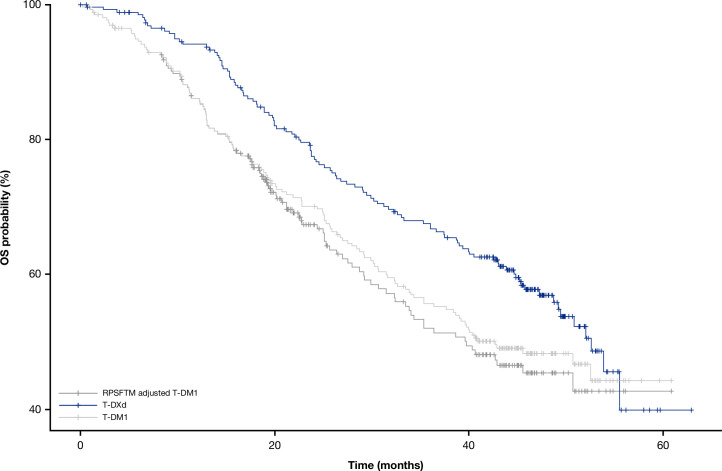
Sensitivity analysis for OS. RPSFTM adjusted Kaplan-Meier curve of OS for the T-DM1 group plotted with the unadjusted OS curves for the T-DXd group and T-DM1 group. OS, overall survival; RPSFTM, rank-preserving structural failure time models; T-DM1, trastuzumab emtansine; T-DXd, trastuzumab deruxtecan.

In the sensitivity analysis (Extended Data Fig. 2), using a median rank-preserving structural failure time model (RPSFTM), the adjusted median OS for the T-DM1 group was 39.8 months (95% CI, 32.4 months to NE). The HR for OS between the T-DXd group and the RPSFTM-adjusted T-DM1 group was 0.66 (95% CI, 0.51–0.87%).

### Safety

Median treatment duration was 18.2 months (range, 0.7–56.6 months) with T-DXd and 6.9 months (range, 0.7–55.2 months) with T-DM1 at the data cutoff. Similar rates of any-grade treatment-emergent adverse events (TEAEs) were observed in both treatment groups (Table 3; 99.6% (256 patients) with T-DXd versus 95.4% (249 patients) with T-DM1). Grade ≥3 TEAEs occurred in 149 T-DXd–treated patients (58.0%) and 136 T-DM1–treated patients (52.1%), of which 48.6% and 42.5%, respectively, were drug-related. In the T-DXd and T-DM1 groups, 58 patients (22.6%) and 19 patients (7.3%), respectively, discontinued treatment due to drug-related TEAEs. In the T-DXd group, the most common drug-related TEAEs associated with discontinuation were pneumonitis (6.6% (17 of 257)) and interstitial lung disease (ILD) (5.4% (14 of 257)). In the T-DM1 group, the most common drug-related TEAEs associated with discontinuation were pneumonitis (1.5% (four of 261)) and platelet count decrease (1.5% (four of 261)) (Extended Data Table 2). Drug-related TEAEs associated with dose reduction occurred in 72 patients (28.0%) with T-DXd and in 40 patients (15.3%) with T-DM1, and drug-related TEAEs leading to drug interruption occurred in 113 patients (44.0%) and 48 patients (18.4%), respectively (Table 3).

**Table 3:** Overall safety summary

*n* (%)	T-DXd, 5.4 mg/kg Q3W, *n* = 257	T-DM1, 3.6 mg/kg Q3W, *n* = 261
**Any-grade TEAEs**	256 (99.6)	249 (95.4)
Drug-related	252 (98.1)	228 (87.4)
**Grade** ≥**3 TEAEs**	149 (58.0)	136 (52.1)
Drug-related	125 (48.6)	111 (42.5)
**Serious TEAEs**	71 (27.6)	59 (22.6)
Drug-related	35 (13.6)	20 (7.7)
**TEAEs leading to drug discontinuation**	63 (24.5)	27 (10.3)
Drug-related	58 (22.6)	19 (7.3)
**TEAEs leading to dose reduction**	73 (28.4)	40 (15.3)
Drug-related	72 (28.0)	40 (15.3)
**TEAEs leading to drug interruption**	146 (56.8)	78 (29.9)
Drug-related	113 (44.0)	48 (18.4)
**TEAEs associated with death**	9 (3.5)	7 (2.7)
Drug-related	0	0

Exposure-adjusted incidence rates (EAIRs) were measured to account for differences in treatment duration between the T-DXd and T-DM1 groups. EAIRs for any-grade TEAEs per patient-year were 0.53 with T-DXd and 1.10 with T-DM1 (Extended Data Table 3). EAIRs for grade ≥3 TEAEs were 0.31 and 0.60, and EAIRs for serious TEAEs were 0.15 and 0.26 with T-DXd and T-DM1, respectively. The most common TEAEs (reported in ≥20% of patients) were similar between the current and previous data cutoff analyses (Extended Data Table 4)^23^.

Adjudicated drug-related ILD and/or pneumonitis occurred in 43 patients (16.7%) in the T-DXd group and in nine patients (3.4%) in the T-DM1 group during the entire study period through the 20 November 2023 data cutoff (Table 4). In the T-DXd group, 11 patients (4.3%) had a grade 1 event, 30 patients (11.7%) had a grade 2 event and two patients (0.8%) had a grade 3 event. Since the previous data cutoff (25 July 2022), four new adjudicated drug-related ILD events (all grade 2) were reported with T-DXd. In the T-DM1 group, five patients (1.9%) had a grade 1 event, three patients (1.1%) had a grade 2 event and one patient (0.4%) had a grade 3 event. No grade 4 or 5 events of ILD or pneumonitis were reported in either treatment group. In the T-DXd group, any-grade adjudicated drug-related ILD was reported in 14 patients (5.4%) within 6 months of the first dose, in 12 patients (4.6%) between 6 and 12 months, in 11 patients (4.2%) between 12 and 24 months and in six patients (2.3%) after 24 months (Extended Data Table 5). EAIRs for ILD and pneumonitis were 0.09 with T-DXd and 0.04 with T-DM1.

**Table 4:** Adjudicated drug-related ILD and pneumonitis^a,b^

*n* (%)	T-DXd, 5.4 mg/kg Q3W, *n* = 257	T-DM1, 3.6 mg/kg Q3W, *n* = 261
Any grade	43 (16.7)	9 (3.4)
Grade 1	11 (4.3)	5 (1.9)
Grade 2	30 (11.7)	3 (1.1)
Grade 3	2 (0.8)	1 (0.4)
Grade 4	0	0
Grade 5	0	0
Grade ≥3	2 (0.8)	1 (0.4)

^a^The grade is based on the worst Common Terminology Criteria for Adverse Events (CTCAE) grade within the same adverse or ILD event.

^b^There were four new events (all grade 2) reported since the previous data cutoff (25 July 2022) with a time to onset of 832 d (not recovered or resolved), 851 d (recovered or resolved with sequelae), 910 d (recovered or resolved with sequelae) and 961 d (recovered or resolved).

Left ventricular dysfunction or left ventricular ejection fraction (LVEF) decrease occurred in 11 patients (4.3%) in the T-DXd group and in four patients (1.5%) in the T-DM1 group. Since the previous data cutoff, there were two events of left ventricular dysfunction or LVEF decrease (one grade 1 event in the T-DXd group and one grade 2 event in the T-DM1 group). EAIRs were 0.02 for both the T-DXd and T-DM1 groups.

## Discussion

In this updated analysis of the DESTINY-Breast03 phase 3 clinical trial in patients with previously treated HER2-positive metastatic breast cancer, T-DXd continued to demonstrate clinically meaningful improvement in efficacy compared with T-DM1 and a manageable safety profile that was consistent with previous results^23^. The median PFS and ORR by investigator assessment reinforced the clinical benefit of T-DXd over T-DM1 and were consistent with the analysis at the previous data cutoff^23^. Median OS was reached in both treatment groups in this updated analysis, with an approximate 10-month improvement over T-DM1 observed with T-DXd and a reduction in the risk of death by approximately 27%, which has not been previously observed in this setting.

The ORR by investigator assessment reported in the T-DXd group in the current analysis was consistent with the ORR by BICR and by the investigator with T-DXd reported in the previous analysis^23^. However, there were differences in the number of complete responses reported in the T-DXd group by the investigator in this analysis (12.6%, *n* = 33) and in the previous analysis (11%, *n* = 30) compared with that reported by BICR in the previous data cutoff analysis (21%, *n* = 55), possibly indicating that the investigators were more conservative in declaring a complete response^23^. Responses appeared to be more durable with T-DXd treatment, with a median DoR by investigator assessment of 30.5 months (median follow-up, 43.0 months) in the T-DXd group compared with 17.0 months (median follow-up, 35.4 months) in the T-DM1 group.

The clinical benefit of T-DXd over T-DM1 in this updated data cutoff is evidenced by the improved median PFS, which was approximately four times longer with T-DXd at 29.0 months than with T-DM1 at 7.2 months, consistent with the previous analysis^23^. Furthermore, almost half of the patients (45.7%) in the T-DXd group were progression free at 3 years and more than 40% (41.5%) of patients were progression free at 4 years (however, several patients were censored at that time point). The median PFS in the T-DXd group was longer than the median PFS reported with first-line pertuzumab, trastuzumab and docetaxel combination therapy at the end-of-study analysis of the CLEOPATRA trial (18.7 months), and the PFS rate was below 40% at 4 years in that trial^9^; however, these cross-trial comparisons should be interpreted cautiously given the continuously changing treatment landscape of HER2-positive metastatic breast cancer. The median PFS2 by investigator assessment with T-DXd was approximately twice as long as that with T-DM1 in this updated analysis, which suggests that patients may derive a better clinical benefit when treated with T-DXd before T-DM1.

To our knowledge, the median OS with T-DXd in DESTINY-Breast03 is the longest reported OS in this disease setting (median OS, 52.6 months; median follow-up, 43 months). This is in the range of the CLEOPATRA trial in the first-line setting, which demonstrated a median OS of 57.1 months (median follow-up, 99.9 months) at the end-of-study analysis in patients with HER2-positive metastatic breast cancer treated with pertuzumab, trastuzumab and docetaxel combination therapy^9^. The median OS observed in the T-DM1 group in DESTINY-Breast03 was 42.7 months, which is longer than that reported in the EMILIA trial (29.9 months)^24^. However, cross-trial comparisons should be interpreted with caution, as the differences observed in median OS between the trials may be due to variations in study design and post-trial therapies used. Since the EMILIA trial was completed, several therapies have been approved for the treatment of HER2-positive metastatic breast cancer^25^. Treatment crossover was not part of the study design of DESTINY-Breast03; however, patients received a range of systemic therapies after the trial and after progression (Table 3). These therapies included other anti-HER2 agents (beyond trastuzumab, T-DM1, T-DXd and pertuzumab), such as HER2-directed tyrosine kinase inhibitors (48.0% for T-DM1) and new HER2-targeted agents (11.6% for T-DM1), which may have impacted OS in the T-DM1 group.

In the post-trial (clinical) setting, 64 patients (32.3%) in the T-DM1 group subsequently received T-DXd. Notably, when adjusting the OS of these patients in the T-DM1 group who received T-DXd after the trial in a sensitivity analysis, the approximate OS improvement with T-DXd versus T-DM1 was >1 year (adjusted median OS of 39.8 months with T-DM1). The efficacy of T-DXd following progression on T-DM1 was previously demonstrated in the DESTINY-Breast02 (NCT03523585) trial (median OS of 39.2 months with T-DXd versus 26.5 months with treatment of physician’s choice)^26^. When taking the data from these two studies together, the better outcomes demonstrated by T-DXd in the present study, including ORR, DoR, PFS and OS, support the potential benefit of T-DXd when used in earlier treatment settings.

Overall, drug-related TEAEs associated with drug discontinuation, dose reduction and drug interruption continued to be higher with T-DXd than with T-DM1, as observed in previous analyses^22,23^. Although more patients in the T-DXd group discontinued treatment due to drug-related TEAEs than in the T-DM1 group, more patients remained on T-DXd treatment than T-DM1 at this updated data cutoff. The safety profile of T-DXd continued to be manageable in this longer-term follow-up of DESTINY-Breast03. Incidence rates of any-grade, grade ≥3 and serious TEAEs were slightly higher with T-DXd than with T-DM1, consistent with reports from previous analyses^22,23^. The median duration of treatment was more than 2.5 times longer with T-DXd than with T-DM1; however, EAIRs, which account for differences between treatment duration, were lower with T-DXd than with T-DM1 for any-grade TEAEs, grade ≥3 TEAEs and serious TEAEs. No new safety signals were observed with long-term treatment, supporting the favorable benefit–risk profile of T-DXd versus T-DM1 in previously treated HER2-positive metastatic breast cancer.

With the additional follow-up since the previous analysis^23^, four new ILD and/or pneumonitis events occurred in the T-DXd group (all grade 2). Most new events resolved or resolved with sequalae (75%) and occurred during the third year of treatment (time to onset between 832 and 961 d). As previously reported^23^, only two patients had grade 3 events in the T-DXd group (both events resolved); no grade 4 or 5 events were observed. Consistent with a previous study, most ILD and/or pneumonitis events occurred within the first year of T-DXd treatment (Extended Data Table 5)^27^. In the T-DM1 group, rates of ILD and pneumonitis increased from 3% to 3.4% at this updated data cutoff; there was only one additional grade 1 event compared with the previous data cutoff^23^. These results support continuous patient monitoring and prompt management of potential ILD and/or pneumonitis when symptoms are detected in patients treated with T-DXd.

Potential limitations of the DESTINY-Breast03 trial have been published^22,23^. In the current analysis, PFS, ORR and DoR were assessed by the investigators, not by BICR; consequently, no formal statistical comparisons were made. We report median OS for both the T-DXd and T-DM1 groups, with an HR supporting improved OS with T-DXd treatment; however, this was an exploratory analysis. Longer follow-up is needed to determine a more precise estimate of the median OS in the T-DXd group due to the number of patients censored; more mature data are expected at the next data cutoff as the study continues.

This long-term analysis reinforces the superiority of T-DXd over T-DM1 in patients with metastatic breast cancer previously treated with taxane and trastuzumab, with the longest median OS reported in this disease setting and more than two-thirds (67.6%) of patients still alive at 3 years. The clinically meaningful improvement in efficacy was consistent with the previous data cutoff. The safety profile of T-DXd continues to be manageable with no cumulative toxicities observed with longer follow-up. Analyses on the impact of T-DXd on long-term responders across studies and exploring the efficacy of T-DXd in the earlier metastatic breast cancer setting (DESTINY-Breast09, NCT04784715) are ongoing.

## Methods

### Trial design

Details of the DESTINY-Breast03 (NCT03529110) study design have been published^22,23^. In summary, this was an open-label, multicenter, phase 3 trial conducted to compare T-DXd with T-DM1 in patients with HER2-positive, unresectable or metastatic breast cancer who were previously treated with trastuzumab and taxane. Patients were randomly assigned 1:1 to receive either T-DXd at 5.4 mg per kg or T-DM1 at 3.6 mg per kg intravenously every 3 weeks. Patients were stratified based on hormone receptor status, prior pertuzumab treatment and history of visceral disease via a web-based system.

Eligible patients had received prior treatment with trastuzumab and taxane, either in an advanced or metastatic setting or with progression that occurred within 6 months of post-neoadjuvant or adjuvant therapy, and had confirmed HER2 positivity according to the American Society of Clinical Oncology–College of American Pathologists guidelines assessed by a central laboratory. Patients were considered to have HER2-positive disease if the tumor was IHC 3+ or IHC 2+ with a positive in situ hybridization result^28^. Documented evidence of radiologic progression either during or after recent treatment or within 6 months after adjuvant therapy was required. Patients were included only if they had adequate renal and hepatic function. Patients with notable or uncontrollable cardiovascular disease, such as recent myocardial infarction, symptomatic heart failure, abnormal troponin levels, prolonged QT intervals or an LVEF below 50% within 28 d of randomization were excluded from the study. Patients previously treated with any HER2-directed antibody–drug conjugate or patients with a history of (noninfectious) ILD and/or pneumonitis requiring steroids or current or unconfirmed ILD and/or pneumonitis were ineligible for the study. Patients with inactive brain metastases or asymptomatic brain metastases that did not require treatment with corticosteroids or anticonvulsants or who had recovered from the acute toxic effect of radiotherapy were eligible for inclusion. A minimum of 2 weeks must have elapsed between the end of whole-brain radiotherapy and study enrollment.

Randomization of patients involved balanced block randomization with a 1:1 allocation ratio for T-DXd and T-DM1. Due to distinct administration protocols and adverse event profiles of the treatments, blinding of patients and investigators was not possible. However, tumor assessments were performed by BICR, which were previously reported^22,23^.

Baseline study assessments preceded the first treatment, followed by assessments on day 1 of each 21-d cycle, including additional evaluations on days 8 and 15 of the first cycle. Tumor assessments occurred every 6 weeks from randomization, irrespective of the treatment cycle. End-of-treatment assessments were conducted within 7 d of discontinuation, with a follow-up at 40 d after treatment or before new anticancer treatment. Subsequent long-term visits were scheduled every 3 months until death, consent withdrawal, loss to follow-up or study closure.

### Trial oversight

The trial was designed by Daiichi Sankyo. Before initiation of the study, the trial protocol was approved by the ethical bodies or institutional review boards at each site. The study was conducted in accordance with the standards set by the Declaration of Helsinki, the International Conference on Harmonization Guideline for Good Clinical Practice, any local regulations and the study protocol. All participating patients provided their informed consent in writing before enrollment. Patients did not receive any compensation for participating in the study.

### Endpoints

The primary endpoint was PFS assessed by BICR^22,23^. The key secondary endpoint was OS. Other secondary and exploratory endpoints reported in this study included ORR, DoR, PFS, PFS2 by investigator assessment and safety. PFS2 was defined as the time from the date of randomization to the first documented progression on the next line of therapy or death due to any cause, whichever occurred first. The next line of therapy was defined as the first new systemic antineoplastic therapy initiated after discontinuation of study treatment regardless of the reason for end of treatment.

### Safety

Adverse events were graded based on Common Terminology Criteria for Adverse Events version 5.0 and coded according to the Medical Dictionary for Regulatory Activities version 25.0. Suspected ILD and/or pneumonitis events were adjudicated by an external independent adjudication committee. Patients with suspected ILD and/or pneumonitis had treatment interrupted until further evaluation, and ILD and/or pneumonitis events were carefully monitored until complete resolution, including after drug discontinuation.

### Sensitivity analysis

The RPSFTM with recensoring techniques was applied to the DESTINY-Breast03 median OS to calculate the estimated acceleration factor exp(ψ) for OS. A hypothetical OS was derived for patients in the T-DM1 group using the estimated acceleration factor exp(ψ) = 0.425; this represents the OS that would have been observed if T-DXd treatment had not been administered after the trial. The sensitivity analysis adhered to the stratified Cox proportional hazards model, which incorporates stratification factors such as hormone receptor status, prior pertuzumab treatment and history of visceral disease, as identified by the interactive response technology platform.

### Statistical analysis

The study aimed to enroll approximately 500 patients, with random assignment determined using EAST software version 6.4. Efficacy analysis was conducted on the full analysis set and included all patients who were randomly assigned to a treatment group. The safety analysis was conducted on the safety analysis set and included all randomly assigned patients who received at least one dose of T-DXd or T-DM1. Analysis of PFS and OS between treatment groups employed a stratified log-rank test, considering randomization factors. This involved presenting Kaplan–Meier survival estimates and curves, including median event times and two-sided 95% CIs (Brookmeyer and Crowley method). Kaplan–Meier estimates at specified intervals with 95% CIs were also provided. HRs and 95% CIs were calculated using a stratified Cox proportional hazards model. The median follow-up duration for OS and its two-sided 95% CI were calculated for each treatment group using the Kaplan–Meier method by reversing the OS censoring and event indicators. Based on a prespecified hierarchical testing procedure, OS (the key secondary endpoint) was tested if PFS by BICR (the primary efficacy endpoint) was statistically significant^22,23^. The current updated OS analysis was exploratory because the prespecified threshold for statistical significance was reached at the second OS interim analysis, although the median OS was not reached previously.

Cochran–Mantel–Haenszel tests, stratified by randomization factors, were used to evaluate ORR. Estimates of ORR were presented with 95% CIs (Clopper–Pearson method). The DoR included median event times and 95% CIs (Brookmeyer and Crowley method), along with Kaplan–Meier estimates. Statistical analysis used SAS version 9.3 or later and R 4.2.0 for the RPSFTM.

### Reporting summary

Further information on research design is available in the Nature Portfolio Reporting Summary linked to this article.

## Online content

Any methods, additional references, Nature Portfolio reporting summaries, source data, extended data, supplementary information, acknowledgements, peer review information; details of author contributions and competing interests; and statements of data and code availability are available at 10.1038/s41591-024-03021-7.

### Supplementary information

Supplementary Information Supplementary Tables 1 and 2

Reporting Summary

## Figures and Tables

**Extended Data Table 1:**
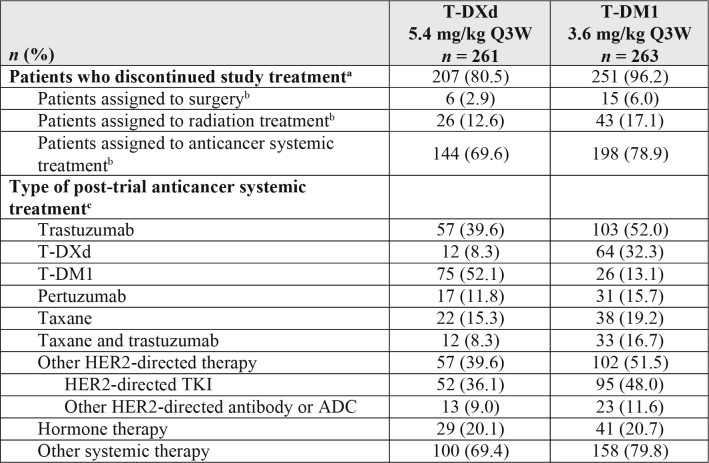
Post-trial anticancer systemic treatment

ADC, antibody–drug conjugates; Q3W, every 3 weeks; T-DM1, trastuzumab emtansine; T-DXd, trastuzumab deruxtecan; TKI, tyrosine kinase inhibitors.

^a^The denominator for calculating the percentage was the number of patients who received at least 1 dose of study treatment (safety analysis set) in the T-DXd or T-DM1 group.

^b^The denominator for calculating the percentage was the number of patients who discontinued study treatment in the T-DXd or T-DM1 group.

^c^The denominator for calculating the percentage was the number of patients who were assigned to any anticancer systemic treatment in the T-DXd or T-DM1 group. Patients could have received more than one type of therapy.

**Extended Data Table 2:**
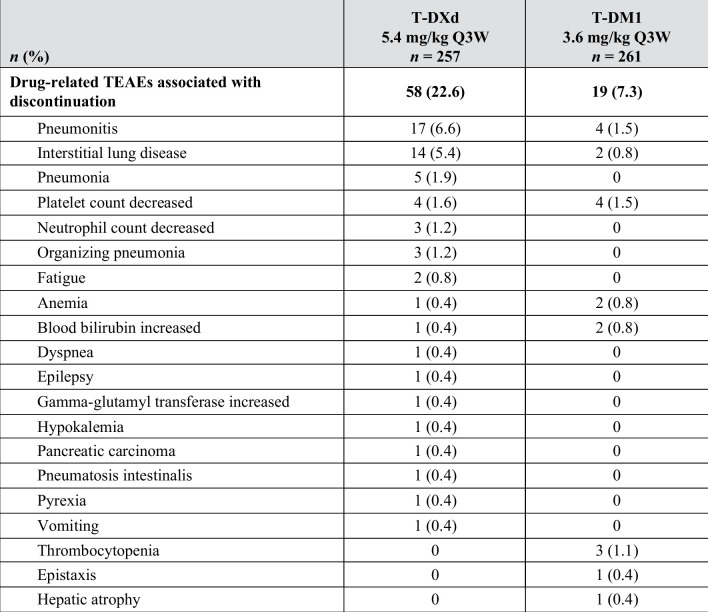
Any-grade drug-related TEAEs associated with study drug discontinuation by preferred term

Q3W, every 3 weeks; T-DM1, trastuzumab emtansine; T-DXd, trastuzumab deruxtecan; TEAE, treatment-emergent adverse event.

**Extended Data Table 3:**
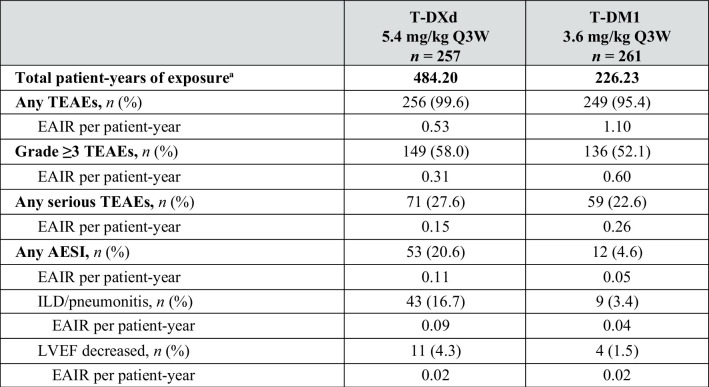
Exposure-adjusted incidence rates

AESI, adverse event of special interest; EAIR, exposure adjusted incidence rates; ILD, interstitial lung disease; LVEF, left ventricular ejection fraction; Q3W, every 3 weeks; T-DM1, trastuzumab emtansine; T-DXd, trastuzumab deruxtecan; TEAE, treatment-emergent adverse event.

^a^Patient-years of exposure are the treatment duration with year as unit.

**Extended Data Table 4:**
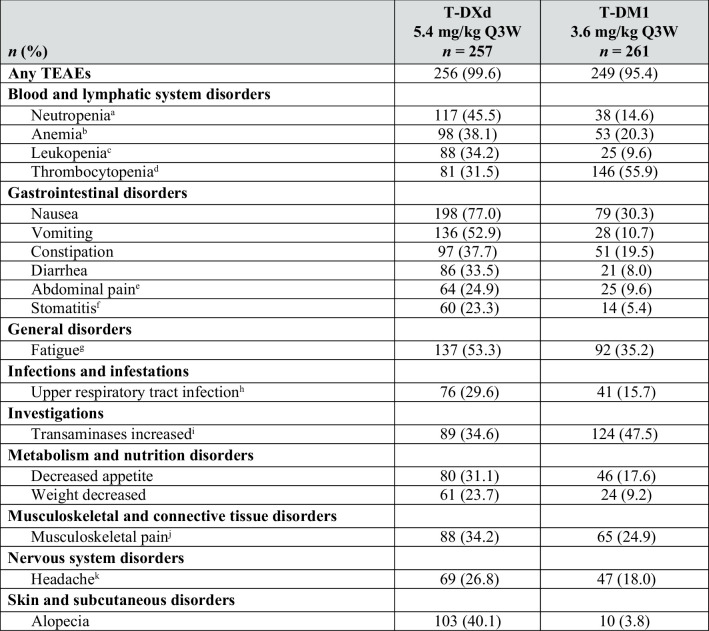
Any-grade TEAEs reported in ≥20% of patients

Q3W, every 3 weeks; T-DM1, trastuzumab emtansine; T-DXd, trastuzumab deruxtecan; TEAE, treatment-emergent adverse event.

^a^Includes the preferred terms neutrophil count decreased and neutropenia.

^b^Includes the preferred terms hemoglobin decreased, red blood cell count decreased, anemia, and hematocrit decreased.

^c^Includes the preferred terms white blood cell count decreased and leukopenia.

^d^Includes the preferred terms platelet count decreased and thrombocytopenia.

^e^Includes the preferred terms abdominal discomfort, abdominal pain, abdominal pain lower, abdominal pain upper, and gastrointestinal pain.

^f^Includes the preferred terms stomatitis, aphthous ulcer, mouth ulceration, oral mucosa erosion, oral mucosal blistering, and oral mucosal eruption.

^g^Includes the preferred terms fatigue, asthenia, malaise, and lethargy.

^h^Includes the preferred terms influenza, influenza like illness, and upper respiratory tract infection.

^i^Includes the preferred terms transaminases increased, aspartate aminotransferase increased, alanine aminotransferase increased, gamma-glutamyltransferase increased, liver function test abnormal, hepatic function abnormal, and liver function test increased.

^j^Includes the preferred terms back pain, myalgia, pain in extremity, musculoskeletal pain, muscle spasms, bone pain, neck pain, musculoskeletal chest pain, and limb discomfort.

^k^Includes the preferred terms migraine, headache, and sinus headache.

**Extended Data Table 5:**
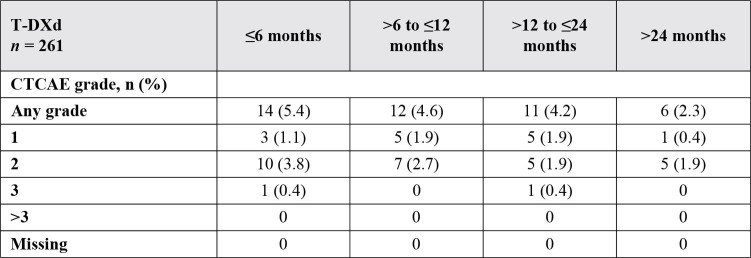
Time to first adjudicated drug-related ILD^a^ in the T-DXd group by CTCAE grade at the time of diagnosis

CTCAE, common terminology criteria for adverse events; ILD, interstitial lung disease; T-DM1, trastuzumab emtansine; T-DXd, trastuzumab deruxtecan.

^a^Time to first adjudicated ILD onset (months) = (onset date of first ILD adjudicated as drug-related - first dose date + 1)/365.25×12.

## Data Availability

Anonymized individual participant data on completed studies and applicable supporting clinical study documents may be available upon request at https://vivli.org/. In cases where clinical study data and supporting documents are provided pursuant to our company policies and procedures, Daiichi Sankyo Companies will continue to protect the privacy of the company and our clinical study participants. Details on data sharing criteria and the procedure for requesting access can be found at this web address: https://vivli.org/ourmember/daiichi-sankyo/. Additional information can be found in the Supplementary Information.
