# Supplementary material for: Trastuzumab deruxtecan versus trastuzumab emtansine in HER2-positive metastatic breast cancer: long-term survival analysis of the DESTINY-Breast03 trial
Source: Nat Med. 2024 Jun 2;30(8):2208–15. doi: 10.1038/s41591-024-03021-7 (PMC11333275; doi:10.1038/s41591-024-03021-7)
Supplement: Supplementary file 1 — Supplementary Tables 1 and 2 [file 41591_2024_3021_MOESM1_ESM.pdf]

nature medicine

Supplementary information https://doi.org/10.1038/s41591-024-03021-7

Trastuzumab deruxtecan versus trastuzumab emtansine in HER2-positive metastatic breast cancer: long-term survival analysis of the DESTINY-Breast03 trial

In the format provided by the authors and unedited

Supplementary Information

Table of Contents

Additional data sharing information Page 2

List of investigational sites Page 3

Additional data sharing information

Data Sharing Questions	Answers
Will individual participant data be available (including data dictionaries)?	Yes
What data in particular will be made available?	Anonymized individual participant data (IPD) on completed studies and applicable supporting clinical trial documents may be available upon request at https://vivli.org/. In cases where clinical trial data and supporting documents are provided pursuant to our company policies and procedures, Daiichi Sankyo, Inc., will continue to protect the privacy of our clinical trial participants. Details on data sharing criteria and the procedure for requesting access can be found at this web address: https://vivli.org/ourmember/daiichi-sankyo/.
What other documents will be available?	Clinical Trial Protocol, Statistical Analysis Plan, Informed Consent Form, and Clinical Study Report. In cases where clinical trial data and supporting documents are provided pursuant to our company policies and procedures, Daiichi Sankyo will continue to protect the privacy of our clinical trial participants.
When will data be available (start and end dates)?	Anonymized IPD will be available when indication supported receives marketing approvals and study results are published.
To whom will data be available?	Qualified science and medical researchers upon formal request and submission of research proposal detailing planned analyses.
For what type of analyses?	De-identified IPD and relevant clinical trial documents will be shared for the purpose of conducting legitimate research as specified in an approved formal research proposal.
By what mechanism will data be made available?	De-identified IPD will be available upon request at https://vivli.org/.

List of investigational sites

Country, Site
Australia
Box Hill Hospital
Peninsula and South Eastern Haematology & Oncology Group
Peter MacCallum Cancer Centre
Princess Alexandra Hospital
St John of God Subiaco Hospital
The Tweed Hospital
Belgium
UZ Leuven
AZ Sint-Lucas
CHU UCL Namur
Universitair Ziekenhuis Brussel
Institut Jules Bordet
UZA
Brazil
A. C. Camargo Cancer Center
CEPHO – Centro de Estudos e Pesquisas de Hematologia e Oncologia
Clínica de Neoplasias Litoral Ltda.
COI – Clínicas Oncológicas Integradas
Hospital Nossa Senhora da Conceição
NOB – Núcleo de Oncologia da Bahia
Clínica de Pesquisas e Centro de Estudos em Oncologia Ginecológica e Mamária Ltda.
ICESP – Instituto do Câncer do Estado de São Paulo Octavio Frias de Oliveira
Canada
Alberta Health Services – Tom Baker Cancer Centre
Toronto Sunnybrook Hospital
China
Cancer Hospital Chinese Academy of Medical Sciences
Fudan University Shanghai Cancer Center
Harbin Medical University Cancer Hospital
Liaoning Cancer Hospital & Institute
Sun Yat-sen Memorial Hospital, Sun Yat-sen University
Sun Yat-sen University, Cancer Center
The First Hospital of Jilin University
Zhejiang Cancer Hospital
Tianjin Medical University Cancer Institute & Hospital
Sir Run Run Shaw Hospital Xiasha Branch, Zhejiang University, School of Medicine
Beijing Hospital
West China Hospital, Sichuan University

Country, Site
France
Hôpital Saint-Louis
Institut Régional du Cancer de Montpellier
Hôpital Nord – CHU Marseille
Centre Hospitalier Lyon Sud
Clinique Victor Hugo – Centre Jean Bernard
Centre Georges François Leclerc
Centre Paul Strauss
Institut Curie – site de Paris
Institut Gustave Roussy
CRLCC Eugene Marquis
Hôpital d’Instruction des Armees Begin
CHU Besançon – Hôpital Jean Minjoz
Institut Sainte Catherine
CARIO – Centre Armoricain de Radiothérapie, Imagerie médicale et Oncologie
Centre Léon Bérard
ICO – Site René Gauducheau
Centre François Baclesse
Hopital Tenon
Centre René Huguenin
ICO – Site Paul Papin
Institut Bergonié
Centre de cancerologie les Dentellieres
Germany
Universitaetsklinikum Erlangen
Universitaetsklinikum Duesseldorf AoeR
Haematologisch-Onkologische Schwerpunktpraxis
Klinikum der Universitaet Muenchen – Campus Grosshardern
Klinikum rechts der Isar der TU Muenchen
Marienhospital Bottrop gGmbH
Universitaetsklinikum Schleswig-Holstein – Campus Luebeck
Rotkreuzklinikum Muenchen gGmbH
Hong Kong
Chinese University of Hong Kong
The University of Hong Kong
Italy
Azienda Ospedaliera Città della Salute e della Scienza di Torino
Azienda Socio Sanitaria Territoriale di Monza (Presidio San Gerardo)
Ospedale San Raffaele
Istituto Clinico Humanitas

Country, Site
IEO Istituto Europeo di Oncologia
A.O.U. Policlinico di Modena
Azienda Ospealiera della Provincia di Lecco
Azienda Ospedaliera Ospedali Riuniti Papardo-Piemonte
Azienda Ospedaliera Universitaria Arcispedale Sant'Anna
Azienda Ospedaliera Universitaria Policlinico Sant'Orsola Malpighi
Azienda Ospedaliero Universitaria di Parma
Azienda Socio Sanitaria Territoriale Papa Giovanni XXIII (Presidio Papa Giovanni XXIII)
Fondazione IRCCS Policlinico San Matteo
IRCCS Centro di Riferimento Oncologico
Istituto Nazionale per la Ricerca sul Cancro di Genova
Istituto Nazionale Tumori Fondazione G. Pascale
Japan
National Cancer Center Hospital
Cancer Institute Hospital of JFCR
Aichi Cancer Center Hospital
Kanagawa Cancer Center
NHO Shikoku Cancer Center
NHO Kyushu Cancer Center
Shizuoka Cancer Center
NHO Hokkaido Cancer Center
NHO Osaka National Hospital
Niigata Cancer Center Hospital
Osaka International Cancer Institute
Saitama Cancer Center
Kumamoto University Hospital
Hiroshima City Hiroshima Citizens Hospital
Okayama University Hospital
Showa University Hospital
Center Hospital of the National Center for Global Health and Medicine
Republic of Korea
Seoul National University Hospital
Samsung Medical Center
Seoul National University Bundang Hospital
Asan Medical Center
Severance Hospital, Yonsei University
National Cancer Center
Ajou University Hospital
Spain
Hospital Universitario Virgen del Rocio

Country, Site
Hospital Infanta Cristina
Hospital Quironsalud Barcelona
Hospital Universitario Virgen Macarena
Hospital Universitario Vall d’Hebron
Hospital Universitario Ramon y Cajal
ICO l’Hospitalet – Hospital Duran i Reynals
MD Anderson Cancer Centre
Hospital Universitario Clinico San Carlos
Hospital Clinico Universitario Virgen de la Victoria
Hospital Clinic de Barcelona
Hospital General Universitario Gregorio Marañon
Hospital Universitario de Canarias
Complejo Hospitalario Universitario A Coruña
Complejo Hospitalario Universitario de Santiago
Hospital Universitario Puerta de Hierro Majadahonda
Hospital Universitario 12 de Octubre
Taiwan, Republic of China
China Medical University Hospital
Koo Foundation Sun Yat-Sen Cancer Center
National Cheng Kung University Hospital
National Taiwan University Hospital
Taipei Veterans General Hospital
United Kingdom
Nottingham University Hospitals City Campus
Royal Surrey County Hospital
Queen Mary University of London
Western General Hospital
University College London Hospitals
Royal Devon and Exeter Hospital (Wonford)
Aberdeen Royal Infirmary
Sarah Cannon Research Institute UK
The Christie
Guy’s Hospital
United States
Dana-Farber Cancer Institute
Allegheny General Hospital
Sharp Memorial Hospital
MD Anderson Cancer Center
The Oncology Institute of Hope and Innovation
North Shore Hematology Oncology Associates, PC

Country, Site
UPMC Cancer Center
University of Cincinnati Medical Center
Piedmont Cancer Institute, PC
University of California San Francisco
UT Southwestern Medical Center
Dayton Physicians, LLC
Millennium Oncology
MultiCare Health System Institute for Research and Innovation
Norton Cancer Institute PARENT
The Ohio State University Wexner Medical Center
UCLA Hematology Oncology (Main Site
University of Nebraska Medical Center
Wake Forest University Baptist Medical Center
Washington Cancer Institute
Health Midwest Ventures Group, Inc d/b/a HCA MidAmerica Division, LLC
Seidman Cancer Center
The Sarah Cannon Research Institute
University of Texas Health Science Center at San Antonio
Tennessee Oncology, PLLC Chattanooga
Vanderbilt University Medical Center
Loyola University Health System
Houston Methodist Cancer Center
University of Rochester
Florida Cancer Specialists-Broadway
Florida Cancer Specialists NORTH
