# Supplementary material for: Trastuzumab deruxtecan versus trastuzumab emtansine in HER2-positive metastatic breast cancer: long-term survival analysis of the DESTINY-Breast03 trial
Source: Nat Med. 2024 Jun 2;30(8):2208–15. doi: 10.1038/s41591-024-03021-7 (PMC11333275; doi:10.1038/s41591-024-03021-7)
Supplement: Supplementary file 2 — Reporting Summary [file 41591_2024_3021_MOESM2_ESM.pdf]

nature portfolio

Corresponding author(s): Javier Cortés

Last updated by author(s): April 17, 2024

Reporting Summary

Nature Portfolio wishes to improve the reproducibility of the work that we publish. This form provides structure for consistency and transparency in reporting. For further information on Nature Portfolio policies, see our Editorial Policies and the Editorial Policy Checklist.

Please do not complete any field with "not applicable" or n/a. Refer to the help text for what text to use if an item is not relevant to your study.
For final submission: please carefully check your responses for accuracy; you will not be able to make changes later.

Statistics

For all statistical analyses, confirm that the following items are present in the figure legend, table legend, main text, or Methods section.

n/a	Confirmed	
☐	☑	The exact sample size (*n*) for each experimental group/condition, given as a discrete number and unit of measurement
☐	☑	A statement on whether measurements were taken from distinct samples or whether the same sample was measured repeatedly
☐	☑	The statistical test(s) used AND whether they are one- or two-sided<br>*Only common tests should be described solely by name; describe more complex techniques in the Methods section.*
☑	☐	A description of all covariates tested
☑	☐	A description of any assumptions or corrections, such as tests of normality and adjustment for multiple comparisons
☐	☑	A full description of the statistical parameters including central tendency (e.g. means) or other basic estimates (e.g. regression coefficient) AND variation (e.g. standard deviation) or associated estimates of uncertainty (e.g. confidence intervals)
☑	☐	For null hypothesis testing, the test statistic (e.g. *F*, *t*, *r*) with confidence intervals, effect sizes, degrees of freedom and *P* value noted<br>*Give P values as exact values whenever suitable.*
☑	☐	For Bayesian analysis, information on the choice of priors and Markov chain Monte Carlo settings
☑	☐	For hierarchical and complex designs, identification of the appropriate level for tests and full reporting of outcomes
☑	☐	Estimates of effect sizes (e.g. Cohen's *d*, Pearson's *r*), indicating how they were calculated

Our web collection on statistics for biologists contains articles on many of the points above.

Software and code

Policy information about availability of computer code

Data collection	Data were collected using Medidata Classic Rave® 2023.2.0.
Data analysis	SAS version 9.3 or later for statistical analysis; R version 4.2.0 for the Rank Preserving Structural Failure Time Model (RPSFTM). EAST software version 6.4 for randomization.

For manuscripts utilizing custom algorithms or software that are central to the research but not yet described in published literature, software must be made available to editors and reviewers. We strongly encourage code deposition in a community repository (e.g. GitHub). See the Nature Portfolio guidelines for submitting code & software for further information.

Data

Policy information about availability of data

All manuscripts must include a data availability statement. This statement should provide the following information, where applicable:

- Accession codes, unique identifiers, or web links for publicly available datasets
- A description of any restrictions on data availability
- For clinical datasets or third party data, please ensure that the statement adheres to our policy

Anonymized individual participant data (IPD) on completed studies and applicable supporting clinical study documents may be available upon request at https://vivli.org/. In cases where clinical study data and supporting documents are provided pursuant to our company policies and procedures, Daiichi Sankyo Companies will continue to protect the privacy of the company and our clinical study subjects. Details on data sharing criteria and the procedure for requesting access can be found at this web address: https://vivli.org/ourmember/daiichi-sankyo/. Additional information can be found in the supplement.

Research involving human participants, their data, or biological material

Policy information about studies with human participants or human data. See also policy information about sex, gender (identity/presentation), and sexual orientation and race, ethnicity and racism.

Reporting on sex and gender	Table 1 of the manuscript reports the sex of the patients. Patient sex data were transferred from patient medical source documents to electronic medical records by the treating physician. No sex- or gender-based analyses were included in this manuscript as there was only 1 male included in each treatment group (Table 1)
Reporting on race, ethnicity, or other socially relevant groupings	Race is reported in Table 1 of the manuscript. There were no race-, ethnicity- or socially relevant-based analysis in this manuscript. Race data were transferred from patient medical source documents to electronic medical records by the treating physician.
Population characteristics	Population characteristics are provided in Table 1 of the manuscript.
Recruitment	Recruitment criteria are availale in the Protocol V.7 (pg.46-50) and have been reported previously: Cortés et al. N Engl J Med. 2022;386:1143-1154 and Hurvitz et al. Lancet. 2023;401:105-117.
Ethics oversight	Independent ethics committees or institutional review boards at each site (full list of investigational sites is provided in the supplementary information) reviewed and approved the protocol as described in the Trial Oversight section of the manuscript. All patients provided written informed consent before enrollment. Patients did not receive compensation for participation in the study.

Note that full information on the approval of the study protocol must also be provided in the manuscript.

Field-specific reporting

Please select the one below that is the best fit for your research. If you are not sure, read the appropriate sections before making your selection.

☑ Life sciences ☐ Behavioural & social sciences ☐ Ecological, evolutionary & environmental sciences

For a reference copy of the document with all sections, see nature.com/documents/nr-reporting-summary-flat.pdf

Life sciences study design

All studies must disclose on these points even when the disclosure is negative.

Sample size	The study aimed to enroll approximately 500 patients. The study was planned with a group sequential design, which included an interim assessment for PFS using a Haybittle-Peto stopping boundary. Sample size was calculated to ensure the study was adequately powered to detect a clinically meaningful PFS benefit; assuming a median PFS of 9.6 months in the T-DM1 arm based on the results of the EMILIA study, it was hypothesized that treatment with T-DXd would result in a HR of 0.7, a 30% reduction in the HR of PFS that would correspond to a 43% improvement in mPFS. For more information please refer to Study Protocol V7.0 (Sample Size Determination - pq.106-108) and the manuscript (Statistical Analysis).
Data exclusions	None
Replication	N/A; this was an exploratory analysis from a randomized clinical trial so there were no replicates.
Randomization	Patients were randomized into 1 of the 2 treatment groups (T-DXd or T-DM1) in a 1:1 ratio. For more information please refer to Study Protocol V7.0 (Randomization - pg.71) and the manuscript (Trial Design).
Blinding	This is an open-label study and it is not feasible to blind treatment allocations for individual patients because of different administration protocols and different adverse event (AE) profiles between T-DXd and T-DM1 therapy. For more information please refer to Study Protocol V7.0 (Blinding - p.51) and manuscript (Trial Design).

Behavioural & social sciences study design

All studies must disclose on these points even when the disclosure is negative.

Study description	
Research sample	
Sampling strategy	
Data collection	
Timing	
Data exclusions	
Non-participation	
Randomization	

Ecological, evolutionary & environmental sciences study design

All studies must disclose on these points even when the disclosure is negative.

Study description

Research sample

Sampling strategy

Data collection

Timing and spatial scale

Data exclusions

Reproducibility

Randomization

Blinding

Did the study involve field work? ☐ Yes ☐ No

Field work, collection and transport

Field conditions

Location

Access & import/export

Disturbance

Reporting for specific materials, systems and methods

We require information from authors about some types of materials, experimental systems and methods used in many studies. Here, indicate whether each material, system or method listed is relevant to your study. If you are not sure if a list item applies to your research, read the appropriate section before selecting a response.

Materials & experimental systems

n/a	Involved in the study	
☑	☐	Antibodies
☑	☐	Eukaryotic cell lines
☑	☐	Palaeontology and archaeology
☑	☐	Animals and other organisms
☐	☑	Clinical data
☑	☐	Dual use research of concern
☑	☐	Plants

Methods

n/a	Involved in the study	
☑	☐	ChIP-seq
☑	☐	Flow cytometry
☑	☐	MRI-based neuroimaging

Antibodies

Antibodies used

Validation

Eukaryotic cell lines

Policy information about cell lines and Sex and Gender in Research

Cell line source(s)	
Authentication	
Mycoplasma contamination	
Commonly misidentified lines (See ICLAC register)	

Palaeontology and Archaeology

Specimen provenance	
Specimen deposition	
Dating methods	

☐ Tick this box to confirm that the raw and calibrated dates are available in the paper or in Supplementary Information.

Ethics oversight	

Note that full information on the approval of the study protocol must also be provided in the manuscript.

Animals and other research organisms

Policy information about studies involving animals; ARRIVE guidelines recommended for reporting animal research, and Sex and Gender in Research

Laboratory animals	
Wild animals	
Reporting on sex	
Field-collected samples	
Ethics oversight	

Note that full information on the approval of the study protocol must also be provided in the manuscript.

Clinical data

Policy information about clinical studies

All manuscripts should comply with the ICMJE guidelines for publication of clinical research and a completed CONSORT checklist must be included with all submissions.

Clinical trial registration	NCT03529110
Study protocol	Study protocol is provided with submission of this manuscript and has been published as supplementary material in Cortes J et al. NEJM 386:1143 and Hurvitz S et al. 2023 Lancet 401:105.
Data collection	From July 20, 2018, to June 23, 2020, 699 patients were screened for eligibility in the trial. 524 patients were enrolled at 169 centers in 15 countries. Data were collected and transferred from patient medical source documents to electronic medical records by the treating physicians. Data were analyzed and interpreted by the sponsor and authors.
Outcomes	The primary endpoint was PFS by BICR and the key secondary endpoint was OS, and were predefined in the protocol (along with other secondary/exploratory endpoints). The efficacy endpoints were based on central assessments, unless otherwise stated. After the outcome measures were reached for the primary endpoint and other endpoints by BICR, the assessments by BICR were discontinued. The primary endpoint was published previously (Cortés et al. N Engl J Med. 2022;386:1143-1154 and Hurvitz et al. Lancet. 2023;401:105-117). The current updated analysis is exploratory. For more information please refer to Study Protocol V7.0 (EFFICACY ASSESSMENTS - p.78 and STATISTICAL METHODS p. 97).

Dual use research of concern

Policy information about dual use research of concern

Hazards

Could the accidental, deliberate or reckless misuse of agents or technologies generated in the work, or the application of information presented in the manuscript, pose a threat to:

No	Yes	
☑	☐	Public health
☑	☐	National security
☑	☐	Crops and/or livestock
☑	☐	Ecosystems
☑	☐	Any other significant area

Experiments of concern

Does the work involve any of these experiments of concern:

No	Yes	
☑	☐	Demonstrate how to render a vaccine ineffective
☑	☐	Confer resistance to therapeutically useful antibiotics or antiviral agents
☑	☐	Enhance the virulence of a pathogen or render a nonpathogen virulent
☑	☐	Increase transmissibility of a pathogen
☑	☐	Alter the host range of a pathogen
☑	☐	Enable evasion of diagnostic/detection modalities
☑	☐	Enable the weaponization of a biological agent or toxin
☑	☐	Any other potentially harmful combination of experiments and agents

Plants

Seed stocks

Novel plant genotypes

Authentication

ChIP-seq

Data deposition

☐ Confirm that both raw and final processed data have been deposited in a public database such as GEO.

☐ Confirm that you have deposited or provided access to graph files (e.g. BED files) for the called peaks.

Data access links
May remain private before publication.

Files in database submission

Genome browser session
(e.g. UCSC)

Methodology

Replicates

Sequencing depth

Antibodies

Peak calling parameters

Data quality

Software

Flow Cytometry

Plots

Confirm that:

- ☐ The axis labels state the marker and fluorochrome used (e.g. CD4-FITC).
- ☐ The axis scales are clearly visible. Include numbers along axes only for bottom left plot of group (a 'group' is an analysis of identical markers).
- ☐ All plots are contour plots with outliers or pseudocolor plots.
- ☐ A numerical value for number of cells or percentage (with statistics) is provided.

Methodology

Sample preparation	
Instrument	
Software	
Cell population abundance	
Gating strategy	

☐ Tick this box to confirm that a figure exemplifying the gating strategy is provided in the Supplementary Information.

Magnetic resonance imaging

Experimental design

Design type	CT or MRI scans of the chest, abdomen, pelvis, and any other sites of disease were performed according to local methods at each study site. For more information please refer to Study Protocol V7.0 (Frequency of Tumor Re-evaluation - pg.136-137 and Method of Assessment - pg.130).
Design specifications	
Behavioral performance measures	

Imaging type(s)	
Field strength	
Sequence & imaging parameters	
Area of acquisition	

Diffusion MRI ☐ Used ☑ Not used

Preprocessing

Preprocessing software	
Normalization	
Normalization template	
Noise and artifact removal	
Volume censoring	

Statistical modeling & inference

Model type and settings	
Effect(s) tested	

Specify type of analysis: ☐ Whole brain ☐ ROI-based ☐ Both

Statistic type for inference

(See Eklund et al. 2016)

Correction

Models & analysis

n/a	Involved in the study
☑	☐ Functional and/or effective connectivity
☑	☐ Graph analysis
☑	☐ Multivariate modeling or predictive analysis

Functional and/or effective connectivity

Graph analysis

Multivariate modeling and predictive analysis

This checklist template is licensed under a Creative Commons Attribution 4.0 International License, which permits use, sharing, adaptation, distribution and reproduction in any medium or format, as long as you give appropriate credit to the original author(s) and the source, provide a link to the Creative Commons license, and indicate if changes were made. The images or other third party material in this article are included in the article's Creative Commons license, unless indicated otherwise in a credit line to the material. If material is not included in the article's Creative Commons license and your intended use is not permitted by statutory regulation or exceeds the permitted use, you will need to obtain permission directly from the copyright holder. To view a copy of this license, visit http://creativecommons.org/licenses/by/4.0/
